# The distinct functions of NODULE INCEPTION-like proteins in nitrate response

**DOI:** 10.1093/plcell/koad038

**Published:** 2023-02-14

**Authors:** Ching Chan

**Affiliations:** Assistant Features Editor, The Plant Cell, American Society of Plant Biologist, USA; Department of Life Science, National Taiwan Normal University, Taipei 11677, Taiwan

**Figure 1.**
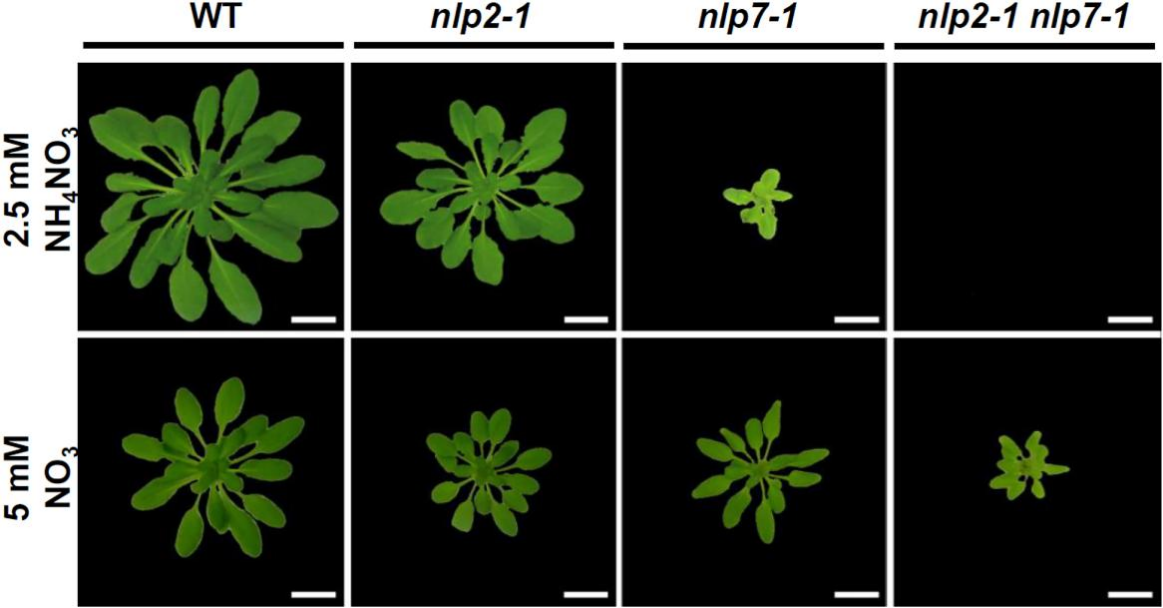
Growth under nonlimiting ammonium nitrate supply distinguishes *nlp2* and *nlp7* mutants. Loss of NLP2 function exacerbated the *nlp7-1* growth defect under ammonium nitrate supply, which was not observed when nitrate was used as the sole nitrogen source. Adapted from Durand et al. (2023), Fig. 8A.

NODULE INCEPTION (NIN)-like proteins (NLPs) are considered master regulators of the nitrate response (Chardin et al. 2014; Konishi and Yanagisawa 2014). NLPs interact with the nitrate-responsive *cis*-elements (NREs), located contiguous to and/or within nitrate-inducible genes (Konishi and Yanagisawa 2013), leading to the activation of nitrate transporters and nitrate/ammonium assimilation enzymes. In general, mutation of NLPs hinders vegetative growth by impairing nitrate-induced gene expression. Apart from transcriptional control, it has recently been observed that NLP7 can directly bind nitrate, as an intra-cellular nitrate sensor (Liu et al. 2022). Nitrate binding residues are present in other Arabidopsis NLPs, and are found on a variety of proteins in other plants as well as bacteria. The functional differentiation between NLP family members, however, remains largely unexplored. Both NLP2 and NLP7 were shown to play major roles in vegetative growth (Konishi et al. 2021). In new work, Mickaël Durand and colleagues (Durand et al. 2023) further dissected NLP2-dependent regulation by deciphering the N-related gene regulatory network. Using transcriptomic and metabolomic analyzes, the authors observed the common roles of NLP2 and NLP7 in mediating the primary nitrate response (PNR), and more importantly, the unique role of NLP2 in connecting the PNR with energy and carbon metabolism under nonlimiting nitrogen supply (see Fig. 1).

NLP2 displayed rapid nuclear retention in response to nitrate, a typical post-translational regulation process known for NLP7 (Marchive et al. 2013). Using chromatin immunoprecipitation, the authors observed significant similarities between NLP2 and NLP7, in terms of binding consensus (NRE) and target genes. An NLP2-dependent regulatory network was proposed, consisting of a cascade of primary targets along with second- and third-tier regulatory components. Notably, significant enrichment of NLP2-bound genes was found in the oxidative pentose phosphate pathway (OPP). The same GO term was also enriched in differentially expressed genes in response to nitrate among NLP2-bound targets, suggesting a specific function of NLP2 in linking nitrogen and carbon metabolism.

Nitrogen and carbon metabolism are closely linked (Vidal et al. 2020). Light and sugar signals in the shoot mediate root growth and nitrate uptake; while an intact OPP is required for subsequent nitrate assimilation (Vidal et al. 2020). In the *nlp2* mutant, untargeted metabolite profiling revealed changes in intermediates of sugar metabolism, which were opposite to or absent in the *nlp7* mutant. Interestingly, such differences were only observed under nonlimiting nitrate conditions, but not under nitrate-limiting conditions. The observation was further supported by the specific downregulation of glycolytic genes in *nlp2*. Therefore, NLP2 is seemingly important in the integration of nitrate assimilation and carbon metabolism in response to nitrate, which distinguishes NLP2 from NLP7 function. This hypothesis was well addressed by a mixed nitrogen supply experiment (see Fig. 1). When nitrate was used as the only nonlimiting nitrogen source, both the *nlp2* and *nlp7* mutants displayed reduced growth, which was additive in the *nlp2 nlp7* double mutant. Strikingly, when equivalent nitrogen was supplied in the form of ammonium nitrate, *nlp2* and *nlp7* showed distinct growth phenotypes. Ammonium nitrate supply partially rescued the *nlp2* growth defect but exacerbated that of *nlp7*. Notably, ammonium nitrate supply to the *nlp2 nlp7* double mutant was lethal.

Taken together, NLP2 and other NLP family members share common features in the transcriptional regulation of the PNR network, but also have distinct functions. NLP2 positively regulates carbon metabolism and energy supply, particularly under nonlimiting nitrate supply. Interestingly, NLP2 and NLP7 physically interact in the nucleus after nitrate resupply (Durand et al. 2023). The tissular distribution of NLPs and their interaction might provide more clues for the specific function and molecular mechanism of NLP2, NLP7, and other NLP family members.
